# The effect of marital status on glioma patient survival: analysis of 617 cases: A SEER-based study

**DOI:** 10.1097/MD.0000000000013900

**Published:** 2018-12-28

**Authors:** Shengrong Long, Mingdong Li, Shaowu Ou, Guangyu Li

**Affiliations:** Department of Neurosurgery, First Affiliated Hospital of China Medical University, Shenyang, China.

**Keywords:** glioma, marital status, Surveillance, Epidemiology, and End Results program, survival analysis

## Abstract

To study the effect of marital status on survival outcome in people diagnosed with glioma, not otherwise specified using the Surveillance, Epidemiology, and End Results (SEER) database.

We chose patients diagnosed with glioma between 2000 and 2014 from the SEER database and recorded their disease-related data. We then analyzed overall 5-year cause-specific survival with respect to different marital statuses. There were 617 patients (262 women and 355 men). Of these, 54.0% (n = 333), 24.6% (n = 152), 8.6% (n = 53), and 12.8% (n = 79) were married, single, divorced (or separated), and widowed, respectively. The 5-year cause-specific survival was 39.30%, 64.50%, 60.40%, and 10.10% in the married, single, divorce (or separated), and widowed groups, respectively. The widowed group had substantially higher risk of glioma-related death than did the married group (hazard ratio 1.77, 95% confidence interval 1.337–2.344, *P* < .001). Being widowed provided higher risk of glioma mortality compared than did marital statuses. Widowed people should be given more support and psychological intervention by society.

## 1 Introduction

Glioma is defined as all tumors derived from neuroepithelial tissue. It is a malignant tumor that originates in the brain and accounts for about half of all intracranial tumors.^[1,2]^ According to the World Health Organization (WHO) tumor grading system, gliomas are classified into WHO I–IV. The higher the level, the higher the degree of malignancy. Among these, grades III and IV have higher malignancy and poor prognosis. Although gliomas are malignant tumors of the brain, not all glioma types grow in a malignant manner.^[3]^ The incidence of glioma in 1973 was approximately 5.9 of 100,000, and it is currently increasing to approximately 6.61 of 100,000. The use of radiodiagnosis is one of the main reasons for the rapid increase in the incidence of glioma.^[1,4]^ At present, the primary treatment strategy for glioma is surgery based, combined with postoperative radiotherapy and chemotherapy. However, overall prognosis remains nonideal.^[5,6]^ For a long time, we have often emphasized that glioma patients should have reasonable medical treatment as biological entities, while ignoring their status as social beings. Socioeconomic status and psychological support also play important roles in their survival prognosis. There has been extensive research on psychological support including marital status for various tumor survival outcomes. It turns out that marital status is an independent predictor of survival prognosis in cancer patients. Married cancer patients have a higher 5-year survival rate than do single, divorced or separated, or widowed patients.^[7–10]^ The current literature has not yet investigated the impact of marital status on the survival of glioma patients. Here, we collected data from the Surveillance, Epidemiology, and End Results (SEER) registration program that met our research criteria. This included individuals diagnosed with glioma between 2000 and 2014. We explored the impact of marital status on glioma patient cause-specific survival (CSS).

## 2 Methods

### 2.1 Data sources

All raw data that met our inclusion and exclusion criteria were from the SEER program, including tumor grade, laterality, year of diagnosis, type, and demographic data, including patient age, sex, and ethnicity. The most recent data we used in this study were based on the incidence—SEER 18 Regs Research Data + Hurricane Katrina Impacted Louisiana Cases, November 2016 Sub (1973–2014 varying). We included patients diagnosed with glioma from 2000 to 2014. Marital status information was divided into 4 categories: married, single, divorced or separated, and widowed.

We included age groups (<50 or ≥50 years old), sex (male or female), race (white, black, other, and unknown), grade (WHO I, II, III, IV), and laterality as an adjustment model covariate. The main survival outcome was survival for specific reasons.

### 2.2 Ethical approval

The current research does not contain any studies with human participants or animals performed by any of the authors.

### 2.3 Inclusion of exclusion criteria

We evaluated patients with glioma from the SEER database using SEER ∗ Stat 8.3.2. Patients were chosen according to the following criteria: age at diagnosis is ≥18 years old; glioma diagnosed between 2000 and 2014; histological types were limited to glioma, not otherwise specified (NOS); marital status was classified as married, divorced, separated, widowed, or single. Patients with unknown survival months or incomplete date information were excluded. The SEER database is a national cancer data sharing project funded by the National Cancer Institute. The project covers approximately 28% of the US population. All case data in this study are from the SEER database.

### 2.4 Data analysis

We compared patient demographics, clinical pathology, and treatment characteristics based on marital status. Survival was expressed by Kaplan-Meier curves and survival differences were compared using a log-rank test. We used univariate and multivariate Cox proportional hazards regression models to calculate hazard ratios (HRs) and their 95% confidence intervals (CIs) to assess survival differences among marital status categories. Variables with significant differences in univariate analysis were entered into a multivariate regression model. All analyses were performed using SPSS version 23.0 statistical software. A *P* value <.05 was considered statistically significant.

## 3 Results

There were 617 patients with a median follow-up of 16 months (range 0–74 months). Of these patients, 54.0% (n = 333) were married, 24.6% (n = 152) were single, 8.6% (n = 53) divorced, and 12.8% (n = 79) were widowed.

Table 1 summarizes the baseline patient characteristics. The proportion of widowed patients among the elderly (≥50 years old) was the highest (96.2% vs 28.9%–66.1%, *P* < .001), female patients had the highest rate of widowhood (75.9% vs 34.2%–56.6%, *P* < .001). Single patients tended to be younger than those with other marital statuses (71.1% vs 3.8%–47.2%, *P* < .001). More male patients were married than single (61.8%–65.8% vs 24.1–43.4%).

**Table 1:**
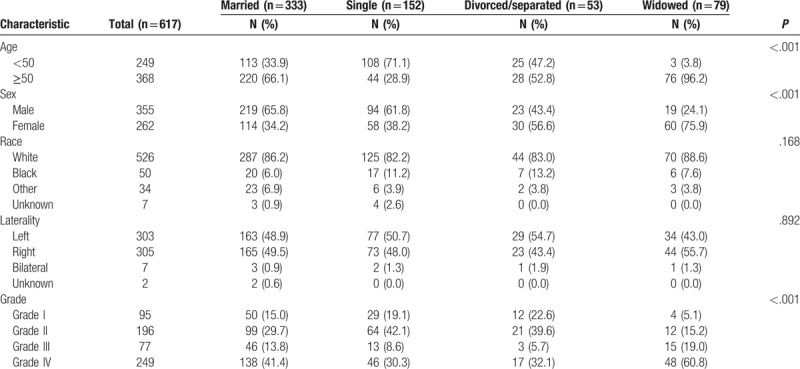
Patient baseline demographic and clinical characteristics.

More female patients tended to be divorced or widowed (56.6%–75.9% vs 34.2%–38.2%). The highest proportion of widowed patients was in grade IV (60.8% vs 30.3%–41.4%). The highest proportion of patients with grade IV in the widowed group (60.8% vs 5.1%–19.0%) (Table 2). Marital status was a prognostic factor for patients with glioma. The overall 5-year glioma CSS was 39.30% in the married group, 10.10% in the widowed group, 65.40% in the single group, and 60.40% in the divorced/separated group.

**Table 2:**
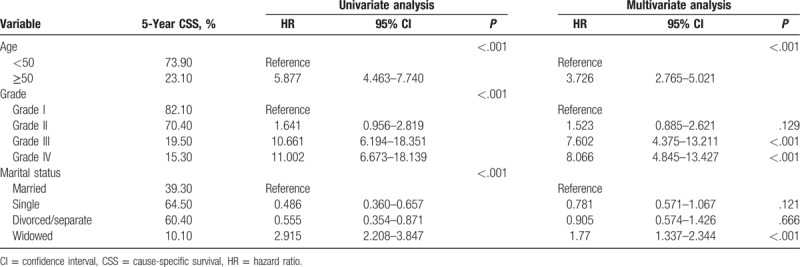
Univariate and multivariate survival analyses of the impact of marital status on glioma cause-specific survival.

Multivariate analysis showed that widowed patients had a higher risk of death than did married patients (HR: 1.77, 95% CI: 1.37–2.344, *P* < .001). However, the risk of death compared to that of single patients (HR: 0.781, 95% CI: 0.571–1.067, *P* = .121) and divorced or separated patients (HR: 0.905, 95% CI: 0.574–1.426, *P* = .666) were not statistically significant.

Other covariates in this and previous studies were shown to be independent predictors of survival prognosis in patients with glioma. Patients older than 50 years of age had a higher risk of death (HR: 3.726, 95% CI: 2.765–5.021) than did patients younger than 50 years of age. Compared with glioma pathological grade I, patients with grade II did not have a significantly higher risk of death (HR: 1.523, 95% CI: 0.885–2.621, *P* = .129). Grade III patients did have a higher risk of death (HR: 7.602, 95% CI: 4.375–13.211, *P* < .001) as did patients with grade IV (HR: 8.066, 95% CI: 4.845–13.427, *P* < .001).

### 3.1 Subgroup analysis of the effect of marital status

#### 3.1.1 Effect of marital status on glioma CSS by tumor WHO grades

**Figure 1:**
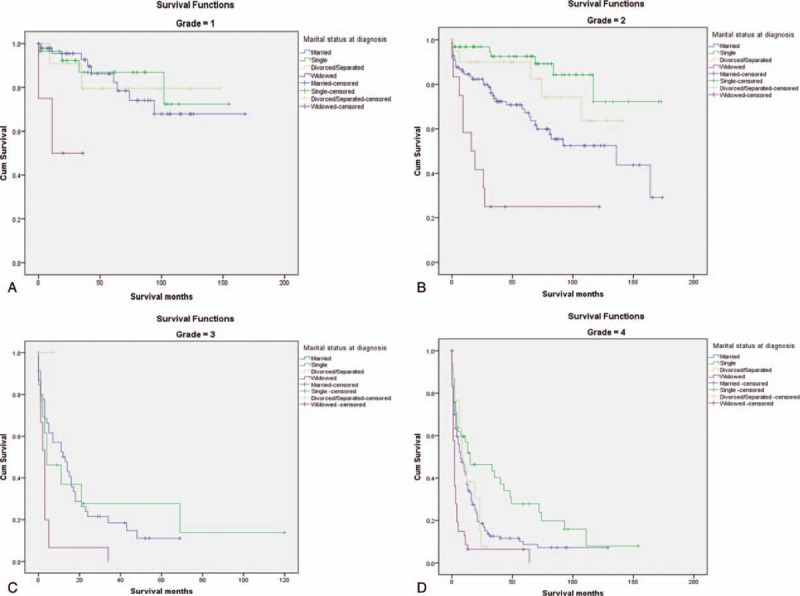
Survival curves of glioma patient according to marital status. A, Grade = 1; *χ*^2^ = 10.2, *P* < .05. B, Grade = 2; *χ*^2^ = 33.744, *P* < .001. C, Grade = 3; *χ*^2^ = 11.244, *P* < .05. D, Grade = 4; *χ*^2^ = 30.596, *P* < .001.

We assessed the impact of various marital statuses on survival outcomes in patients with various pathological grades of glioma. We found that widowed patients had a higher risk of death in each pathological grade (Table 3, Fig. 1). Although single and married/separated patients had a higher 5-year survival rate compared with married patients, multivariate analysis showed no statistical difference.

**Table 3:**
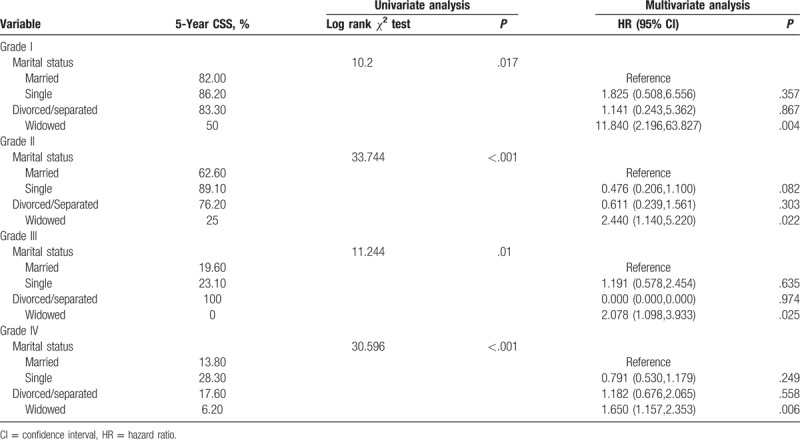
Univariate and multivariate analyses of the impact of marital status on glioma cause-specific survival based on glioma grade.

Compared with married patients, the 5-year survival rate of widowed patients decreased by 32% (50% vs 82%, *P* < .05) for pathological grade I, and the 5-year survival rate decreased by 37.6% for pathological grade II (25% vs 62.60%, *P* < .05). In pathological grade III, the 5-year survival rate decreased by 19.6% (0% vs 19.6%, *P* < .05), and the 5-year survival rate in pathological grade IV decreased by 7.6% (6.20% vs 13.80%, *P* < .05). Multivariate analysis also showed that widowed patients had the lowest 5-year survival rate in all pathological grades of glioma compared with married patients.

### 3.2 Effect of marital status on glioma CSS by patient age

**Figure 2:**
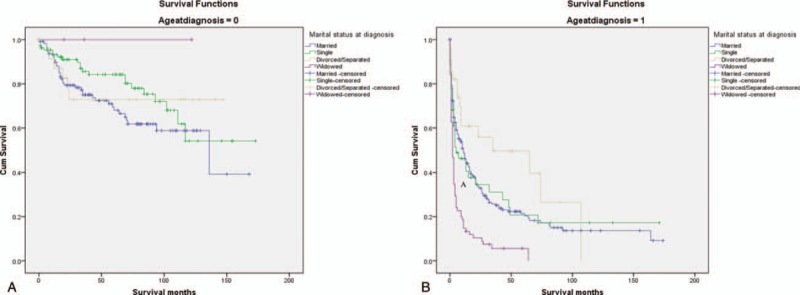
Survival curves of glioma patient according to marital status. A, Age at diagnosis <50; *χ*^2^ = 4.267, *P* = .234. B, Age at diagnosis ≥50; *χ*^2^ = 37.388, *P* < .001.

The patients included in the analysis were divided into 2 groups by using the age cut-off of 50 years, and the survival of glioma patients in various marital statuses was investigated (Table 4, Fig. 2). The results showed that widowed patients had the lowest 5-year survival rate in the group of 50 years or older, and the 5-year survival rate of married patients decreased by 18.4% (6.60% vs 25.00%, *P* < .001). Compared with married glioma patients, the widowed group had a higher risk of death (HR: 1.791, 95% CI: 1.345, 2.384, *P* < .001).

**Table 4:**
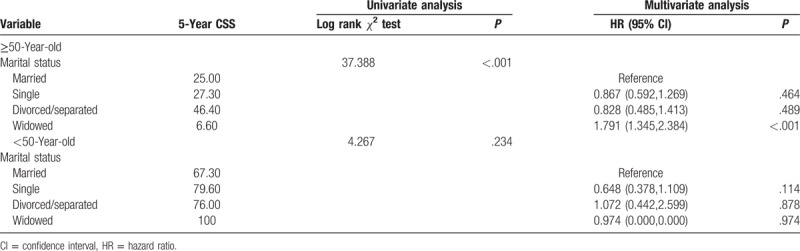
Univariate and multivariate analyses of the impact of marital status on glioma cause-specific survival based on patient age.

## 4 Discussion

We sought to determine the influence of marital status on survival outcome in patients who were diagnosed with glioma between 2000 and 2014 based on a cohort study. We found that marital status was an independent prognostic factor for gliomas after eliminating sex, age, race, grade, and laterality. Widowers had poorer survival than did married patients. As multivariate analysis showed, patients with higher grade tumors and older patients had poorer survival outcome. Race, sex, and laterality of tumor had no significant effect on survival outcomes of patients who were in various marital statuses. In a systematic review, Manzoli found that there was no difference in the effect of marital status on the outcomes in male and female patients.^[11]^ This was consistent with the results of our study.

There was a study that showed that married people were more likely to receive systematic treatment and care services than were widowed patients.^[12,13]^ The differences in the relationship between marital status and mortality may be attributed to several reasons. First, married people have more financial resources, including higher incomes, better employment, and insurance, that can help them access diagnosis and therapy timely. Aizer et al^[13]^ found that people who were with the spouses had a 70% increased chance of follow-up treatment. Spouses encourage patients to be screened for tumors and to seek medical advice as early as possible when they have clinical symptoms. Spouses also encourage patients to undergo active and adequate treatment, increasing the compliance that is critical to cancer treatment. Second, social support also helps improve cancer prognosis.^[13]^ Marriage could provide social positive behavior in a way.^[14]^ In another way, married people are more likely to give up some of their bad habits, including smoking, drugs, and alcoholism because of the sense of responsibility to their family and spouse than are unmarried people. We do not advocate that marriage should be regard as an approach to improve the outcome treatment. However, in the analysis and comparison of the risk of death due to tumor characteristics in marital status, we did not find that prognosis of single patients was worse than that of married patients, possibly related to the insufficient sample size of this study or the insufficient statistical efficacy of the statistics test. However, single, divorced, and widowed people can help themselves by maintaining strong social networks and being able to rely on friends and family for some support.

Many studies have suggested that increased stress and decreased psychosocial support might do harm to immune responses and give rise to progression of tumor by regulating the hypothalamus-pituitary-adrenal axis. Stress could regulate the release of glucocorticoids and catecholamines, further affecting tumor microenvironment and finally contribute to the survival of tumor.^[15–19]^ Chronic psychological stress can promote secretion of cortisol that downregulates the level of cortisol in white blood cells. Stress affects the cellular responses to inflammatory signals^[20]^ and induces excessive cytokine-mediated inflammatory processes that are related to progression of cancer.^[12,13]^

### 4.1 Limitations

First, SEER only recorded marital status at the time of diagnosis. We cannot know whether their marital status after diagnosis changed. Second, as a retrospective study, it is inevitable and possible to introduce some confounding factors into the study. Third, there may be some people who are not legally married, but actually lived in same-sex or heterosexual relationships. In addition, the SEER database lacks detailed information on radiotherapy, chemotherapy, and recurrence of recurrence.^[21]^ Furthermore, our study results were limited to the US population and are not representative of the global population. Last but not least, in the era of individualized medicine, the targeted treatment of glioma has been included in routine treatment. Mutations in driver genes, such as *MGMT*, *IDH1*, and others affect the choice of treatment and the different evaluation of prognosis. In view of these limitations, the results should be interpreted with caution.

## 5 Conclusions

There was an insignificant difference in overall survival from glioma in terms of sex or laterality. Being widowed increased the risk of glioma NOS mortality, compared with glioma patients who are married. Nevertheless, widowed patients could look for support and care from social contacts instead of a marital relationship in some way. Above all, clinicians should pay more attention to patients’ marital status who were diagnosed with glioma when providing individualized treatment. Widows should be given psychosocial support to increase their motivation for treatment, and perhaps their survival and prognosis will be improved as a result.

## Author contributions

Conception and design: S.L.; development of methodology: S.L.; collection and assembly of data: M.L.; data analysis and interpretation: M.L., S.O.; writing, review, and/or revision of the manuscript: all authors; administrative, technical, or material support: G.L.; study supervision: G.L.; final approval: all authors.

**Conceptualization:** Shengrong Long.

**Data curation:** Shengrong Long.

**Formal analysis:** Shengrong Long.

**Funding acquisition:** Guangyu Li.

**Investigation:** Mingdong Li.

**Methodology:** Guangyu Li.

**Project administration:** Shaowu Ou.

**Resources:** Mingdong Li, Shaowu Ou.

**Software:** Shengrong Long.

**Supervision:** Mingdong Li.

**Validation:** Shaowu Ou.
